# Work–Family Conflict, Parental Mental Health, and Children’s Emotional and Behavioral Difficulties

**DOI:** 10.3390/children13020289

**Published:** 2026-02-19

**Authors:** Vitória Dias, Sara Albuquerque, Ana Beato, Stephanie Alves

**Affiliations:** HEI-Lab: Digital Human-Environment Interaction Labs, Lusófona University, Campo Grande 376, 1749-024 Lisbon, Portugal; p8883@ulusofona.pt (V.D.); sara.albuquerque@ulusofona.pt (S.A.); ana.beato@ulusofona.pt (A.B.)

**Keywords:** work–family conflict, parental stress, depressive symptoms, parental self-efficacy, children’s emotional and behavioral difficulties

## Abstract

**Highlights:**

**What are the main findings?**

- Greater work-family conflict is related to higher levels of parent-reported children’s emotional and behavioral difficulties through increased parenting stress and depressive symptoms.
- These associations occurred either among parents of toddlers and preschool-aged children.

**What is the implication of the main finding?**

- Parental mental health promotion may indirectly prevent emotional and behavioral difficulties in young children.
- Family-friendly workplace policies that reduce work–family conflict may support family functioning and child adjustment.

**Abstract:**

Background/Objectives: Work–family conflict (WFC) is a common stressor for working parents and has been associated with poorer child adjustment. However, the mechanisms linking WFC to young children’s emotional and behavioral difficulties remain insufficiently understood. This study examined whether parental mental health mediates the association between WFC and children’s emotional and behavioral difficulties in early childhood. Methods: This quantitative cross-sectional study was conducted in Portugal with 313 parents of children aged 18–72 months. Parents completed validated self-report measures of WFC, parental stress, depressive symptoms, parental self-efficacy, and children’s emotional and behavioral difficulties. Mediation and moderated mediation analyses were performed, testing children’s age (toddlers vs. preschool-aged) as a moderator. Results: Higher WFC was associated with greater emotional and behavioral difficulties in children (parents reported). This association was fully mediated by parental stress and depressive symptoms, whereas parental self-efficacy did not show a significant mediating effect. The indirect pathways were consistent across children’s age groups. Conclusions: The findings indicate that WFC may affect young children’s adjustment, primarily through its impact on parental psychological distress. Supporting parental mental health and reducing WFC may be key targets for early prevention and intervention.

## 1. Introduction

The conciliation of work and family responsibilities represents a central challenge for contemporary families. Over recent decades, structural changes in the labor market have occurred, including increased job demands, longer and less predictable working hours, job insecurity, and the intensification of work, which have placed growing pressure on working parents [1,2]. These demands often collide with family responsibilities, giving rise to work–family conflict (WFC), a form of inter-role conflict in which work demands interfere with family functioning by reducing parents’ available time, energy, and emotional resources for family life [3,4,5].

In Portugal, work–family interference is frequently conditioned by high performance pressure, long working hours, and irregular schedules [6], which are reflected in elevated levels of WFC across different professional groups [6,7,8]. Although legislative measures such as parental leave and flexible working arrangements are available, work-to-family interference remains prevalent when organizational practices fail to adequately support parents [9]. Recent Eurofound data indicate that Portuguese workers report longer and more demanding working patterns compared to the European average, including a higher prevalence of extended working hours and interference between work schedules and family responsibilities [10]. These patterns may contribute to heightened levels of WFC in the Portuguese context.

Empirical evidence consistently shows that higher levels of WFC are associated with increased psychological distress, depressive symptoms, emotional exhaustion, and stress-related health problems [1,2,11,12]. Meta-analytic reviews further indicate that psychosocial work stressors closely related to WFC, such as job strain, low control, effort–reward imbalance, and job insecurity, are robust predictors of common mental health problems [13]. Longitudinal findings suggest that WFC is often not a transient experience but may persist or accumulate over time, with chronic exposure being particularly detrimental to parental mental health [2].

Although the consequences of WFC for adult well-being are well established, family systems are inherently interdependent, and stress experienced by one family member, particularly a parent, may have cascading effects on other members. As such, research has increasingly examined its implications for children’s emotional and behavioral development [4,14,15].

### 1.1. WFC and Children’s Emotional and Behavioral Difficulties

Children’s emotional and behavioral difficulties are commonly conceptualized as internalizing and externalizing problems. Internalizing difficulties involve inwardly directed emotional symptoms, such as anxiety, sadness, and withdrawal, whereas externalizing difficulties refer to outwardly directed behavioral problems, including aggression, hyperactivity, and oppositional behaviors. This distinction is particularly relevant in early childhood, a developmental period marked by rapid changes in emotional and behavioral regulation [16,17]. These dimensions are developmentally significant given their continuity and associations with later mental health problems, academic difficulties, and impaired social functioning [17,18].

A growing body of evidence suggests that parental WFC is associated with higher levels of emotional and behavioral difficulties in children. Cross-sectional and longitudinal studies have documented such associations across parents’ gender and diverse family and cultural contexts. For example, Strazdins and colleagues [4] found that WFC experienced by either mothers or fathers was associated with increased emotional and behavioral symptoms in young children, even after controlling for socioeconomic and family characteristics. Importantly, when both parents experienced high levels of WFC, children exhibited greater difficulties, suggesting additive or cumulative family-level effects. More recently, Yucel and Latshaw [18] reported that higher levels of WFC in both parents were associated with greater emotional problems in children, although associations with conduct problems were primarily observed for mothers. Longitudinal evidence further indicates that sustained exposure to WFC is particularly detrimental, with accumulated exposure across multiple time points being associated with poorer child mental health outcomes [19,20,21].

Evidence also suggests that associations between WFC and child outcomes may vary according to the type of difficulty, with internalizing problems often showing more consistent links than externalizing behaviors [14]. However, although these associations are increasingly documented, the processes through which WFC affects children’s emotional and behavioral adjustment remain to be further clarified.

Children’s emotional and behavioral difficulties are also influenced by a range of sociodemographic and family characteristics. Previous research indicates that factors such as children’s age and sex, family composition (e.g., number of children), family structure, and broader sociocultural contexts, including minority or migrant status, are associated with both parental stress and children’s psychological adjustment. Accordingly, these variables are commonly considered as relevant covariates in studies examining the links between work–family conflict, parental mental health, and child outcomes [17,18,22].

### 1.2. Parental Mental Health as a Central Mechanism

Parental mental health has been identified as a central mechanism through which work–family stress may influence children’s emotional and behavioral adjustment [5,19]. From an ecological and family stress perspective, stressors originating in the work context are expected to influence children primarily through their effects on parents’ psychological functioning and caregiving capacities, rather than through direct exposure [4,23].

In line with this perspective, WFC has been identified as a significant predictor of parental depressive symptoms. Longitudinal evidence indicates that increases in WFC over time are associated with subsequent increases in depressive symptoms, whereas reductions in WFC are linked to improvements in parental mental health [1,2]. Meta-analytic findings further identify depressive symptoms as one of the most consistent mediators linking work–family factors to child mental health outcomes [16]. Empirical studies show that parental depression is associated with higher levels of both internalizing and externalizing problems in children [24,25].

Parental stress represents a closely related but conceptually distinct indicator of parental mental health. Parenting stress refers to the stress experienced in the parenting role when perceived demands exceed available resources [26].

Research consistently shows that higher levels of WFC are associated with increased parental stress [5,27]. In turn, parental stress has been robustly linked to higher levels of emotional and behavioral difficulties in children [22]. Longitudinal evidence suggests that parental stress may dynamically co-evolve with children’s behavioral difficulties over time, reinforcing maladaptive family processes [19]. Also, there is evidence that parental stress often fully or partially mediates the association between WFC and child outcomes. Santos [27] found that parental stress fully mediated the association between both maternal and paternal WFC and children’s externalizing behaviors. Similarly, Vieira et al. [28] reported that WFC was indirectly associated with children’s internalizing and externalizing difficulties through disruptions in parent–child relationship quality. Also, Van den Eynde et al. [5] reported that the association between WFC and child behavior operated indirectly through parental well-being and parenting performance rather than through direct pathways. These findings highlight parental stress as a proximal mechanism linking work-related strain to children’s emotional and behavioral difficulties.

In line with depletion-based models of parental mental health, sustained exposure to high demands and limited resources may progressively compromise parents’ psychological functioning. Recent research further illustrates that cumulative strain and contextual stressors, including WFC, are associated with increased parental exhaustion and reduced psychological resources [29]. These frameworks support the relevance of stress-related and depressive processes as proximal mechanisms through which WFC may affect children’s emotional adjustment.

In addition to distress-based aspects of parental mental health, such as stress and depressive symptoms, parental self-efficacy represents a distinct psychological resource referring to parents’ beliefs in their ability to successfully manage parenting demands and positively influence their child’s development [3,30]. A substantial body of literature links higher parental self-efficacy to better parent–child relationship quality, lower levels of parental depression and stress, and more favorable child developmental outcomes, including fewer behavioral problems and better emotional adjustment [30]. Parental self-efficacy has been associated with child outcomes both directly and indirectly through parenting behaviors [31]. Moreover, recent evidence indicates that parental self-efficacy operates within transactional family processes, functioning both as a predictor and as an outcome associated with positive parenting and better child adjustment [32].

Evidence also suggests that WFC is negatively associated with parental self-efficacy. Adams and Golsch [33] found that higher levels of WFC predicted lower parental self-efficacy over time, particularly among mothers. Cinamon et al. [3] similarly reported that higher WFC was associated with lower perceived parenting competence and poorer perceived quality of parent–child interactions. These findings suggest that persistent interference between work and family roles may undermine parents’ confidence in their parenting abilities.

### 1.3. The Moderating Role of Children’s Age

The potential impact of WFC on children’s emotional and behavioral difficulties is likely to vary as a function of the child’s age, particularly in early childhood [14,15]. From a developmental and ecological perspective, age reflects differences in children’s biopsychosocial dependence on caregivers, as well as in parental demands [11]. During toddlerhood, children rely heavily on parents for emotional co-regulation, behavioral guidance, and basic daily routines, making them particularly sensitive to changes in parental psychological functioning [34,35]. Parental stress, depressive symptoms, or reduced self-efficacy may therefore have more immediate and pronounced effects on toddlers’ emotional and behavioral regulation [22,25].

As children transition into the preschool period, gains in cognitive, emotional, and self-regulatory capacities may reduce direct dependence on parental co-regulation [17,36]. However, this developmental stage is also characterized by increasing behavioral autonomy and emotional complexity, which may interact differently with parental mental health difficulties [22,35]. Thus, while the impact of WFC may remain significant, its manifestations may vary across age, for example in the relative prominence of internalizing versus externalizing difficulties [14,18].

In this sense, children’s age may function both as an indicator of developmental vulnerability and as a proxy for parental demands, potentially moderating the strength of the associations between WFC, parental mental health, and children’s emotional and behavioral difficulties. Despite its theoretical relevance, few studies have explicitly examined age-related differences in these processes within early childhood.

### 1.4. The Present Study

Despite extensive evidence linking WFC to parental mental health and children’s emotional and behavioral difficulties, important gaps remain. Most studies have examined individual mediators in isolation, typically focusing on either parental stress or depressive symptoms, rather than integrating multiple indicators of parental mental health within a single analytical framework [14,19,27]. As highlighted by Bilodeau et al. [14], although indirect effects through parental characteristics are consistently observed, direct associations between WFC and child outcomes are often inconsistent.

Moreover, relatively little research has integrated negative indicators of parental mental health with resource-based constructs such as parental self-efficacy. Given evidence that self-efficacy is closely intertwined with both stress and depression and plays a central role in parenting and child adjustment [30,32], its inclusion may offer a more nuanced understanding of the pathways linking WFC to children’s emotional and behavioral difficulties.

**Figure 1:**
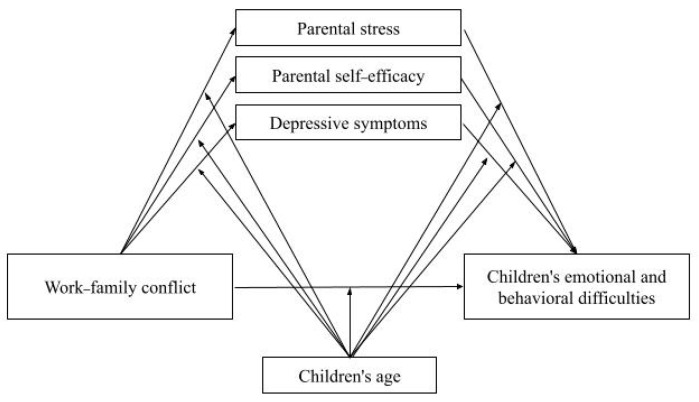
Conceptual model of the study.

This study aimed to analyze the association between WFC and parent-reported children’s emotional and behavioral difficulties and whether this relationship is mediated by parental mental health, considering three distinct indicators: parental stress, depressive symptoms, and parental self-efficacy. The specific objectives were to analyze: (1) the association between WFC and parent-reported children’s emotional and behavioral difficulties; (2) the association between the three indicators of parental mental health and parent-reported children’s emotional and behavioral difficulties; (3) whether the association between WFC and parent-reported children’s emotional and behavioral difficulties is mediated by parental mental health; and (4) whether the proposed mediation is moderated by children’s age (toddler vs. preschool) (see Figure 1). 

## 2. Materials and Methods 

### 2.1. Procedure

This study has a quantitative and cross-sectional design and is part of a larger project entitled *Promoting parental and child mental health: From understanding to intervention*, which was approved by the Ethics and Deontology Committee of the School of Psychology and Life Sciences of Lusófona University (Ref.CEDIC-2024-6-25). Participants were eligible to participate if they: (1) were aged 18 or over; (2) had at least one child aged between 18 and 72 months; (3) could read and understand Portuguese; and (4) lived in Portugal. Participants were recruited through digital media platforms (e.g., Facebook pages about parenting) and educational institutions (e.g., daycare centers, kindergartens) who were asked to share the research protocol’s link with the parents by e-mail. Participants were invited to complete an online questionnaire, stored on the Qualtrics platform, with an expected duration of 40 min. Informed consent was obtained from all participants at the beginning of the online questionnaire, after being presented the nature and goals of the study. Participation was anonymous and voluntary, with no monetary compensation.

### 2.2. Instruments

#### 2.2.1. Sociodemographic Characteristics

A questionnaire was designed by the research team to assess the sociodemographic characteristics of the participants (i.e., age, sex, marital status, professional status of the partner, nationality, ethnicity, education, type of employment, number of hours worked per week, area of residence, number of children, number of household members, and socioeconomic status). Information related to the target children included age, gender, existence of emotional and behavioral problems and, if applicable, whether the child had received or was receiving professional support. Participants were also asked whether they were currently in a marital relationship with the child’s father/mother.

#### 2.2.2. Work–Family Conflict

To assess WFC, the Portuguese version (PV) of the Work–Family Conflict Scale [37] (PV: [38]) was used. This subscale consists of 4 items, answered on a 7-point Likert scale, ranging from 1 (Strongly Disagree) to 7 (Strongly Agree). The total score is obtained by averaging the items and can range from 1 to 7, with higher scores reflecting higher WFC. Regarding internal consistency, both the original version and the Portuguese validation demonstrated good internal consistency [39]. In this study, Cronbach’s alpha was 0.88.

#### 2.2.3. Children’s Emotional and Behavioral Difficulties

To assess parents’ reports of emotional and behavioral difficulties in children between 18 and 60 months of age, the Preschool Pediatric Symptom Checklist (PPSC) [39] (PV: [40]) was used. It consists of 18 items answered on a 3-point Likert scale, ranging from 0 (Not at all) to 2 (Very much). The total score is obtained by summing the items and can range from 0 to 36, with higher scores reflecting higher emotional and behavioral difficulties in children (as perceived by parents) [39,40]. Regarding internal consistency, both the original version and the Portuguese validation showed good internal consistency [39,40]. In this study, Cronbach’s alpha was 0.86.

For children over 60 months old, the Pediatric Symptom Checklist-17 (PSC-17) [41] (PV: [42]) was used. It is composed of 17 items rated on a 3-point Likert scale, ranging from 0 (Never) to 2 (Frequently). The total score is obtained by summing the items and can range from 0 to 34, with higher scores reflecting higher parent-reported children’s emotional and behavioral difficulties. Regarding internal consistency, the original version showed good internal consistency (α = 0.89), and psychometric studies of the Portuguese validation are ongoing. In this study, Cronbach’s alpha was 0.79. In order to use a single variable, the total scores of the PPSC and PSC-17 were standardized (0–100) and a unique score of parent-reported children’s emotional and behavioral difficulties was created.

#### 2.2.4. Parental Stress

To assess parental stress, the Parental Stress Scale (PSS) [43] (PV: [44]) was used. It consists of 18 items, answered on a 5-point Likert scale, ranging from 1 (Strongly disagree) to 5 (Strongly agree). The items include aspects considered to be more positive and aspects considered to be more negative about parenting, with items 1, 2, 5, 6, 7, 8, 17, and 18 being scored inversely [45,46]. The total score is obtained by summing the items and can range from 18 to 90, with higher scores reflecting higher levels of parental stress [46]. Regarding internal consistency, the PSS showed a good internal consistency both in the original version and in the Portuguese validation. In this study, Cronbach’s alpha was 0.84. 

#### 2.2.5. Depressive Symptoms

To assess parents’ depressive symptoms, the depression subscale of the Hospital Anxiety and Depression Scale (HADS) [47] (PV: [48]) was used. It consists of 7 items, answered on a 4-point Likert scale, ranging from 0 (for example, as far as before) to 3 (for example, almost not at all). The total score is obtained by summing the items and can range from 0 to 21, with higher scores reflecting more depressive symptoms [48]. Regarding internal consistency, both the original version and the Portuguese validation showed good internal consistency values [48]. In this study, Cronbach’s alpha was 0.74.

#### 2.2.6. Parental Self–Efficacy

To assess parents’ perceptions of their self-efficacy in parenting, the Parental Self-Efficacy subscale of the Parental Self-Regulation Scale was used (MaaP) [49] (PV: [50]). This subscale consists of four items answered on a five-point Likert scale ranging from 1 (Strongly disagree) to 5 (Strongly agree). The total score is obtained by summing the items and can range from 4 to 20, with higher scores reflecting higher levels of parental self-efficacy [51]. Regarding internal consistency, both the original version and the Portuguese validation showed good internal consistency values. In this study, Cronbach’s alpha was 0.87. 

### 2.3. Statistical Analysis

Data analysis was conducted in IBM SPSS software (v. 28). For the sociodemographic characterization of the sample, descriptive statistics were calculated. Pearson correlations (between continuous variables) and analyses of variance (ANOVA) (between continuous and categorical variables) were calculated between the sociodemographic variables and the main study variables in order to identify potential covariates to be included in the regression models.

To analyze the association between WFC, parental mental health, and children’s emotional and behavioral difficulties, Pearson correlations and hierarchical regression analyses were computed. Correlations were interpreted according to Cohen [52], considering correlations between 0.10 and 0.30 to be weak, associations between 0.30 and 0.50 to be moderate, and associations above 0.50 to be strong. In the hierarchical regression analysis, a model with three blocks was performed: in the first block, the main independent variable (WFC) was introduced; in the second block, the potential mediators (parental stress, depressive symptoms, and parental self-efficacy) were introduced; and, in the third block, the sociodemographic characteristics that were significantly associated with children’s emotional and behavioral difficulties (i.e., nationality, number of children, children’s age, child’s sex, and marital relationship with the father/mother) were introduced. 

To analyze whether parental stress, depressive symptoms, and parental self-efficacy mediated the association between WFC and children’s emotional and behavioral difficulties, a simple mediation model was tested using the PROCESS macro for SPSS (Model 4; [53]). The three dimensions of parental mental health were introduced together, resulting in a single mediation model tested. The mediation model was adjusted for the covariates found to be significant in the final adjusted hierarchical regression model (i.e., nationality, children’s age and sex). Indirect effects were calculated by bootstrapping resampling (5000 samples) and considered significant when zero was not contained between the minimum and maximum of the estimated 95% confidence intervals (CI). Unstandardized regression coefficients were reported [54].

Finally, to assess whether the mediation model occurs equally in parents with toddlers (i.e., children aged between 18 months and 36 months) and preschool-aged children (i.e., children aged between 37 months and 72 months), a moderated mediation model using PROCESS macro for SPSS was computed (Model 59; [53]). Children’s age was recoded into two distinct categories: 0—parents of toddlers and 1—parents of preschool-aged children. The value defined for significance was *p* < 0.05 for all analyses.

## 3. Results

### 3.1. Participants

The sample consisted of 313 participants, whose characteristics are detailed in Table 1. Participants were aged between 20 and 56 years (*M* = 37.35; *SD* = 5.77). Most participants were mothers (84.7%), had completed higher education (70.9%), were working (91.4%), mostly full-time (91.6%), in person (63.3%), and between 5 and 80 h per week (*M* = 38.95; *SD* = 8.17).

Most participants were Portuguese (93.6%), lived in urban areas (82.7%), identified themselves as part of the white ethnic majority (93.9%), and described their family income as average (62.3%). The participants’ households ranged from 1 to 5 members, with an average of 4 individuals, including 1 to 4 children (*M* = 1.60; *SD* = 0.68). Most participants were in a relationship (86.6%) and their partner was employed (94.5%). Of the 313 participants, 26.8% were parents of toddlers, 53.7% were parents of preschool-aged children, and 19.2% had at least one child in both age groups.

Parents with more than one child aged between 18 and 72 months old were asked to report information about the child with whom they felt the greatest difficulties (referred in this study as the target child). Target children had a mean age of 48.33 months (*SD* = 17.14; 18–83), with 46% being male and 54% female. More than half of the participants were in a marital relationship with the father/mother of the target child (87.9%). Only 17.3% of the children had emotional and/or behavioral problems, of which 35.2% received or are currently receiving professional support. 

### 3.2. Preliminary Analyses

Descriptive statistics and correlations between study variables are described in Table 2. Higher levels of WFC were significantly and weakly associated with higher levels of children’s emotional and behavioral difficulties. 

Higher levels of WFC were significantly and moderately associated with higher levels of parental stress and weakly associated with higher levels of depressive symptoms and lower levels of parental self-efficacy. Higher levels of parental stress and depressive symptoms were significantly and moderately associated with higher levels of children’s emotional and behavioral difficulties and higher levels of parental self-efficacy were significantly and weakly associated with fewer levels of children’s emotional and behavioral difficulties.

Parents of non-Portuguese nationality reported higher levels of children’s emotional and behavioral difficulties, *r* = 0.13, *p* < 0.05, as well as parents with more children, *r* = 0.13, *p* < 0.05. Parents of older children, *r* = 0.31, *p* < 0.001, male children, *r* = −0.18, *p* < 0.01, and who are not in a marital relationship with the father/mother of that child, *r* = −0.16, *p* < 0.01, reported higher levels of children’s emotional and behavioral difficulties.

### 3.3. Hierarchical Regression Model

The hierarchical regression analyses are presented in Table 3. The first block, which includes WFC, explained 2.4% of the variance of children’s emotional and behavioral difficulties, *F* (1, 311) = 7.65, *p* < 0.01, with WFC being positively and significantly associated with children’s emotional and behavioral difficulties. The second block, which includes parental stress, depressive symptoms, and parental self-efficacy, explained 18.2% of the variance of children’s emotional and behavioral difficulties *F* (3, 308) = 20.00, *p* < 0.001.

Parental stress and depressive symptoms were significantly and positively associated with children’s emotional and behavioral difficulties. WFC became non-significant. The third block included covariates and explained 12.6% of the variance of children’s emotional and behavioral difficulties, *F* (5, 303) = 16.74, *p* < 0.001. Parental stress and depressive symptoms remained significantly and positively associated with children’s emotional and behavioral difficulties. Parents’ nationality and children’s age were positively and significantly associated with children’s emotional and behavioral difficulties, while children’s sex was negatively and significantly associated with children’s emotional and behavioral difficulties. Parents of non-Portuguese nationality, older children, and whose target child is male reported higher children’s emotional and behavioral difficulties.

### 3.4. The Mediating Role of Parental Mental Health in the Association Between WFC and Children’s Emotional and Behavioral Difficulties

**Figure 2:**
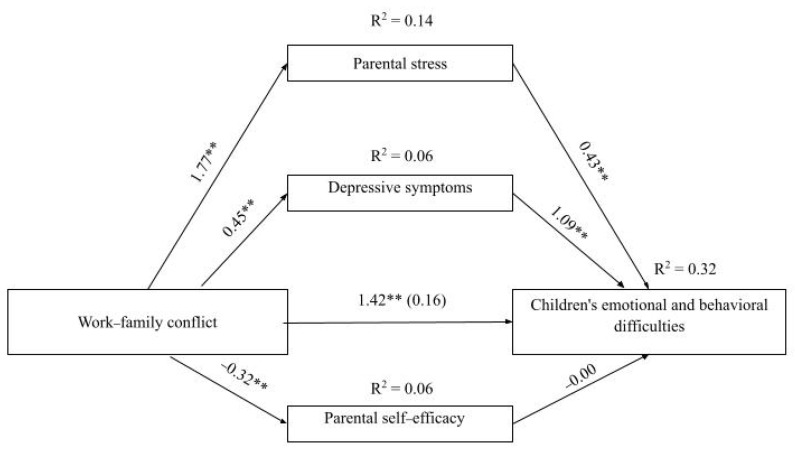
Direct and indirect effects of the relationship between WFC and children’s emotional and behavioral difficulties, through parental stress, depressive symptoms, and parental self-efficacy. Path values represent unstandardized regression coefficients. The total effect of WFC on children’s emotional and behavioral difficulties (before inclusion of the mediators) is described in parentheses and the direct effect (after inclusion of the mediators) is represented by the value outside parentheses. The mediation model was adjusted for covariates (i.e., nationality, children’s age and children’s sex). ** *p* < 0.01.

The mediation model is represented in Figure 2. The total effect of WFC on parent-reported children’s emotional and behavioral difficulties was statistically significant; *b* = 1.421, *SE* = 0.48, 95% CI (0.48, 2.36). Together with the covariates (i.e., nationality, child’s age in months, and child’s sex), WFC explained 17.6% of the variance of children’s emotional and behavioral difficulties *F* (4, 308) = 16.45, *p* < 0.01. The direct effect of WFC (after introducing the mediators) on children’s emotional and behavioral difficulties was not statistically significant; *b* = 0.16, *SE* = 0.47, 95% CI (−0.75/1.08). Taken together, the WFC, the covariates, and the three dimensions of parental mental health explained 32.4% of the variance of children’s emotional and behavioral difficulties; *F* (7, 305) = 20.91, *p* < 0.001. To improve clarity, mediation results are presented for each dimension of parental mental health.

#### 3.4.1. Mediator 1: Parental Stress

Higher levels of WFC were associated with higher levels of parental stress, *b* = 1.77, *SE* = 0.28, 95% CI (1.23/2.32), explaining 13.6% of the variance of parental stress, *F* (4, 308) = 12.11, *p* < 0.001. Parental stress was significantly and positively associated with children’s emotional and behavioral difficulties, *b* = 0.43, *SE* = 0.11, 95% CI (0.22/0.64). The indirect effect of parental stress in the association between WFC and children’s emotional and behavioral difficulties was significant, *b* = 0.77, *SE* = 0.21, 95% CI (0.41/1.22). Higher levels of WFC were associated with higher levels of parental stress which, in turn, were associated with higher levels of children’s emotional and behavioral difficulties.

#### 3.4.2. Mediator 2: Depressive Symptoms

Higher levels of WFC were associated with higher levels of depressive symptoms, *b* = 0.45, *SE* = 0.11, 95% CI (0.23/0.67), explaining 5.9% of the variance of depressive symptoms, *F* (4, 308) = 4.80, *p* < 0.01. Depressive symptoms were significantly and positively associated with children’s emotional and behavioral difficulties, *b* = 1.10, *SE* = 0.25, 95% CI (0.60/1.58). The indirect effect of depressive symptoms in the association between WFC and children’s emotional and behavioral difficulties was significant, *b* = 0.49, *SE* = 0.20, 95% CI (0.16/0.93). Higher levels of WFC were associated with higher levels of depressive symptoms, which, in turn, were associated with higher levels of children’s emotional and behavioral difficulties.

#### 3.4.3. Mediator 3: Parental Self-Efficacy

Higher levels of WFC were associated with lower parental self-efficacy, *b* = −0.32, *SE* = 0.09, 95% CI (−0.50/−0.14), explaining 5.9% of the variance of parental self-efficacy, *F* (4, 308) = 4.80, *p* < 0.01. Parental self-efficacy was not significantly associated with children’s emotional and behavioral difficulties, *b* = −0.00, *SE* = 0.32, 95% CI (−0.63/0.63). The indirect effect of self-efficacy in the association between WFC and children’s emotional and behavioral difficulties was not significant, *b* = −0.00, *SE* = 0.11, 95% CI (−0.23/0.22).

### 3.5. Moderated Mediation Model

As detailed in Table 4, WFC was significantly associated with parental stress, depressive symptoms, and parental self-efficacy. Parental stress and depressive symptoms revealed positive and significant associations with children’s emotional and behavioral difficulties, while parental self-efficacy was not significantly associated with children’s emotional and behavioral difficulties. Children’s age was significantly and positively associated with children’s emotional and behavioral difficulties. The interaction effect between children’s age and each of the main study variables was not statistically significant.

The moderated mediation index values showed that children’s age was not a significant moderator of the mediation models (see Table 5). A significant mediation through parental stress was found for both parents of toddlers and parents of preschool-aged children. Regarding the mediation through depressive symptoms, although the moderated mediation index was not statistically significant, the results suggest a stronger mediation effect for parents of preschool-aged children than for parents of toddlers. The mediation through self-efficacy was not significant for either group.

## 4. Discussion

This study aimed to examine the association between WFC and children’s emotional and behavioral difficulties, as well as to test the mediating role of different indicators of parental mental health—parental stress, depressive symptoms, and parental self-efficacy—in this association and the potential moderating effect of children’s age. Overall, the findings provide a coherent integration of ecological, family stress, and developmental perspectives, suggesting that the association between WFC and children’s adjustment may operate primarily through parents’ psychological functioning rather than through direct pathways.

### 4.1. WFC and Child Emotional and Behavioral Difficulties

Consistent with the first hypothesis, higher levels of WFC were associated with greater emotional and behavioral difficulties in children, as perceived by parents. Although the magnitude of this direct association was modest, the pattern aligns with international evidence showing small but robust associations between parents’ work–family experiences and child’s adjustment outcomes [4,14,15]. These results support the notion that WFC constitutes a relevant contextual risk factor within the child’s developmental ecology, even during early childhood. From a theoretical standpoint, these findings are consistent with the Ecological Systems Theory [55], which posits that stressors arising in one microsystem (work) can spill over into other microsystems (family), indirectly shaping child’s development. As suggested by Van den Eynde et al. [5], the intersection between work and family demands contributes to the emotional and relational climate of the family environment, with implications for children’s functioning over time.

### 4.2. Work–Family Conflict and Parental Mental Health

In line with the second hypothesis, WFC was significantly associated with poorer parental mental health, reflected in higher levels of parental stress and depressive symptoms and lower levels of parental self-efficacy. These findings replicate a substantial body of literature identifying WFC as a chronic stressor that undermines parental psychological well-being [1,2,11].

The particularly strong association between WFC and parental stress suggests that interference between work and family roles is experienced as a direct burden on the parenting role. When parents’ time, energy, and emotional resources are depleted by work demands, parenting tasks may be perceived as more taxing and difficult to manage, increasing stress specifically related to parenting [5,17]. Depressive symptoms, in turn, may reflect a more cumulative and pervasive impact of WFC on parental mental health, consistent with longitudinal evidence indicating that sustained exposure to WFC predicts increases in depressive symptomatology over time [2].

The negative association between WFC and parental self-efficacy is also consistent with previous research [3,33], suggesting that persistent conflict between work and family responsibilities may erode parents’ confidence in their ability to effectively meet their children’s needs. However, as discussed below, the role of parental self-efficacy appears to be more complex when examined alongside other indicators of parental mental health.

### 4.3. Parental Mental Health and Child Emotional and Behavioral Difficulties

Regarding the third hypothesis, parental stress and depressive symptoms were positively and significantly associated with children’s emotional and behavioral difficulties as perceived by parents, whereas parental self-efficacy was not significantly associated with such difficulties when considered simultaneously with the other mental health indicators. This pattern is largely consistent with prior research identifying parental stress and depression as robust predictors of both internalizing and externalizing problems in young children [22,24,25].

The impact of parental stress can be understood within family stress models, which propose that elevated stress compromises parental sensitivity, emotional availability, and consistency in caregiving, thereby increasing children’s vulnerability to emotional and behavioral difficulties [19]. Similarly, parental depressive symptoms are associated with reduced responsiveness, greater irritability, and less adaptive parent–child interactions, all of which have been linked to poorer child’s adjustment [24].

The absence of a significant effect for parental self-efficacy in the final model, despite its significant bivariate association with children’s emotional and behavioral difficulties, warrants careful consideration. One plausible explanation is that when more proximal and potent indicators of parental distress—such as stress and depressive symptoms—are simultaneously included, the unique contribution of parental self-efficacy is attenuated. Recent evidence suggests that parental self-efficacy often operates within transactional processes, being shaped by children’s behavior and exerting its influence indirectly through parenting practices rather than acting as an independent direct predictor of children’s outcomes [32]. Thus, in this study, parental self-efficacy may play a more prominent role in longitudinal or process-oriented models that incorporate parenting behaviors.

### 4.4. The Mediating Role of Parental Mental Health

The mediation analyses provided a central contribution of this study, partially supporting the fourth hypothesis. The association between WFC and children’s emotional and behavioral difficulties was fully mediated by parental stress and depressive symptoms, but not by parental self-efficacy. These findings reinforce the view that the association between WFC and child adjustment may be largely indirect, operating through parents’ psychological functioning [4,14]. Parental stress emerged as the most robust mediator, corroborating previous studies that identify it as a key proximal mechanism linking work-related strain to child outcomes, particularly in families with young children [5,27]. Depressive symptoms also showed a significant indirect effect, extending prior research by highlighting parental depression as an important pathway through which difficulties in balancing work and family demands may translate into concerns about children’s mental health [23,56].

Taken together, these results support an integrated interpretation in which higher levels of WFC are associated with increased parental distress, which, in turn, may undermine the emotional and relational quality of the family environment, heightening children’s risk for emotional and behavioral difficulties.

### 4.5. The Moderating Role of Children’s Age

Children’s age did not significantly moderate the mediation models. Nevertheless, a trend toward a stronger mediating effect of depressive symptoms among parents of preschool-aged children was observed. Although this finding should be interpreted cautiously, it may reflect the fact that preschool-aged children are more attuned to adults’ emotional states and more capable of perceiving parental mood, potentially increasing their sensitivity to parental depressive symptoms [36,57]. The absence of statistically significant moderation effects suggests that, within this sample, the associations between WFC, parental mental health, and child difficulties were relatively similar across toddlerhood and the preschool period. This finding contributes to the literature by indicating that the impact of WFC and parental distress on child adjustment manifests relatively consistently across early childhood, underscoring the importance of early preventive interventions.

### 4.6. Clinical and Preventive Implications

From a practical perspective, the findings underscore the importance of interventions that address parental mental health as a central pathway linking WFC to children’s emotional and behavioral adjustment. Parenting and family-based interventions that incorporate stress-management strategies and early identification of depressive symptoms may have indirect benefits for children’s well-being. At a broader level, the results support the relevance of family-friendly workplace policies, such as flexible working arrangements and organizational support for parents, as preventive strategies with potential downstream effects on child development. Integrating work–family considerations into mental health and child-focused services may therefore enhance the effectiveness of early prevention efforts.

### 4.7. Limitations and Future Directions

Several limitations should be acknowledged. First, the cross-sectional design precludes causal inferences regarding the directionality of the associations, and longitudinal designs are needed to examine transactional and developmental processes over time. Second, the predominance of mothers in the sample limits the generalizability of the findings to fathers and other caregivers, highlighting the need for more gender-balanced samples in future research. Third, the exclusive reliance on parent-reported measures may introduce shared method variance and perceptual biases, particularly for internalizing symptoms. On a related note, while the combination of PPSC and PSC-17 scores into a single variable allowed for analytic comparability, since they capture overlapping but not identical symptom profiles, this should be considered when interpreting results. Future studies would benefit from multi-informant and observational approaches, as well as from the inclusion of objective or contextual indicators of work–family conditions.

## 5. Conclusions

This study provides evidence consistent with the hypothesis that WFC is associated with young children’s emotional and behavioral difficulties, primarily through its links with parental psychological well-being. The findings suggest that parental stress and depressive symptoms may be important mechanisms within this association, while indicating a more indirect role for parental self-efficacy. Overall, supporting parents’ mental health and work–family balance may constitute promising avenues for prevention and early intervention efforts aimed at promoting healthy child development.

## Figures and Tables

**Table 1:** Sociodemographic characteristics (N = 313).

Variables	*n*	%	*M* (*SD*)
Parent Characteristics			
Sex			
Female	265	84.7	
Male	48	15.3	
Age (in years)	313		37.35 (5.77)
Civil Status			
In a relationship	271	86.6	
Not in a relationship	42	13.4	
Partner’s professional status (in a relationship)			
Employee	256	94.5	
Unemployed	15	5.5	
Nationality			
Portuguese	293	93.6	
Other nationality	20	6.4	
Ethnic group			
White	294	93.9	
Other	12	3.8	
Missing	7	2.2	
Education			
With higher education	222	70.9	
Without higher education	91	29.1	
Professional status			
Employee	286	91.4	
Unemployed	27	8.6	
Type of work (employee)			
Full Time	262	91.6	
Part-time	20	6.99	
Missing	4	1.42	
Working method (employee)			
In-person only	181	63.3	
Exclusively in teleworking	25	8.74	
Hybrid	76	26.6	
Missing	4	1.40	
Hours per week (employee)	282		38.95 (8.17)
Missing	4		
Place of residence			
Urban area	259	82.7	
Rural area	54	17.3	
Household	313	3.52 (0.85)	
Socioeconomic status			
Below average	37	11.8	
On average	195	62.3	
Above average	81	25.9	
Number of children	313		1.60 (0.68)
Children’s characteristics			
Children’s age	313		48.33 (17.14)
Children’s Sex			
Female	169	54.0	
Male	144	46.0	
Parenting			
Parents of toddlers	84	26.8	
Parents of preschool-aged children	168	53.7	
Parents of toddlers and preschool-aged children	60	19.2	
Missing	1	0.3	
Emotional and behavioral problems in children			
No	259	82.7	
Yes	54	17.3	
Professional support (yes)			
No	35	64.8	
Yes	19	35.2	
Relationship with father/mother			
Yes	275	87.9	
No	38	12.1	

**Table 2:** Descriptive statistics and correlations between main study variables.

			Correlations				
	*M* (*SD*)	Min/Max	1	2	3	4	5
1. Work–family conflict	3.88 (1.59)	1.00/7.00	---	0.34 ***	0.22 ***	−0.20 **	0.16 **
2. Parental stress	36.32 (8.28)	21.00/62.00	---	---	0.44 ***	−0.50 ***	0.40 ***
3. Depressive symptoms	4.99 (3.23)	0.00/18.00	---	---	---	−0.40 ***	0.36 ***
4. Parental self–efficacy	16.62 (2.59)	8.00/20.00	---	---	---	---	−0.26 ***
5. Children’s emotional and behavioral difficulties	23.80 (14.71)	0.00/73.53	---	---	---	---	---

Note. ** *p* < 0.01; *** *p* < 0.001.

**Table 3:** Association between WFC, parental mental health, and children’s emotional and behavioral difficulties.

Variables	Block 1	Block 2	Block 3
	WFC	Potential Mediators	Covariates
	ΔR^2^ = 0.024 R^2^ Adjusted = 0.021 *b* (SE)	ΔR^2^ = 0.182 R^2^ Adjusted = 0.196 *b* (SE)	ΔR^2^ = 0.126 R^2^ Adjusted = 0.312 *b* (SE)
Constant	18.23 (2.18) ***	2.12 (8.34)	−3.60 (8.58)
Independent variable			
WFC	1.43 (0.52) **	0.02 (0.50)	0.21 (0.47)
Potential mediators			
Parental stress		0.52 (0.11) ***	0.41 (0.11) ***
Depressive symptoms		1.03 (0.27) ***	1.07 (0.25) ***
Parental self-efficacy		−0.14 (0.34)	−0.54 (0.32)
Covariates			
Nationality ^1^			6.93 (2.86) *
Number of children			0.94 (2.86)
Children’s age			0.24 (0.04) ***
Children’s sex ^2^			−5.13 (1.39) ***
Relationship with father/mother ^3^			−3.70 (2.15)

Note. * *p* < 0.05, ** *p* < 0.01; *** *p* < 0.001; WFC—Work–Family Conflict; ^1^ 0 = Portuguese, 1 = Other Nationality; ^2^ 0 = Male, 1 = Female; ^3^ 0 =No, 1 = Yes.

**Table 4:** Results of the mediation-moderated analyses.

	Mediation Model I (Parental Stress)		Mediation Model II (Depressive Symptoms)		Mediation Model III (Parental Self-Efficacy)		Dependent Model I (Children’s Emotional and Behavioral Difficulties)	
	b (95% bootCI)	SE	b (95% bootCI)	SE	b (95% bootCI)	SE	b (95% bootCI)	SE
Constant	2.29 * [−4.50, −0.07]	1.13	−1.06 [−1.96, −0.16]	0.46	1.04 ** [0.32, 1.76]	0.37	24.36 ** [20.57, 28.15]	1.93
WFC	1.79 * [1.25, 2.33]	0.28	0.44 ** [0.22, 0.66]	0.11	−0.31 ** [−0.49, −0.14]	0.09	0.23 [−0.74, 1.20]	0.49
Parental stress	--	--	--	--	--	--	0.48 ** [0.26, 0.70]	0.11
Depressive symptoms	--	--	--	--	--	--	1.04 ** [0.52, 1.55]	0.26
Parental self-efficacy	--	--	--	--	--	--	−0.13 [−0.80, 0.54]	0.34
Children’s age	1.59 [−0.35, 3.53]	0.99	−0.21 [−0.99, 0.58]	0.40	0.37 [−0.99, 0.26]	0.32	6.19 ** [2.91, 9.48]	1.67
WFC X Children’s age	0.49 [−0.70, 1.68]	0.60	0.08 [−0.40, 0.56]	0.25	0.19 [−0.19, 0.58]	0.20	0.91 [−1.20, 3.03]	1.07
Parental stress X Children’s age	--	--	--	--	--	--	−0.06 [−0.58, 0.47]	0.27
Depressive symptoms X Children’s age	--	--	--	--	--	--	0.51 [−0.63, 1.65]	0.58
Parental self-efficacy X Children’s age	--	--	--	--	--	--	−0.62 [−2.17, 0.93]	0.79

Note. WFC—Work–Family Conflict; b refers to the non-standardized coefficient values. In parentheses, the values refer to the lower and upper limits of the 95% Confidence Interval (CI). ST = Standard Error; * *p* <. 05; ** *p* < 0.01.

**Table 5:** Conditional indirect effects.

Moderator	Conditional Indirect Effect	Bootstrap SE	Bootstrap LLCI	Bootstrap ULCI	Moderate Mediation Index
Dependent variable: Children’s emotional and behavioral difficulties; Mediator: Parental stress					
Toddlers ^1^	0.75	0.38	0.14	1.60	
Preschool-aged children ^2^	0.89	0.27	0.41	1.44	
	--	0.47	−0.86	0.96	0.14
Dependent variable: Children’s emotional and behavioral difficulties; Mediator: Depressive symptoms					
Toddlers ^1^	0.25	0.21	−0.07	0.74	
Preschool-aged children ^2^	0.55	0.27	0.12	1.15	
	--	0.34	−0.34	1.00	0.29
Dependent variable: Children’s emotional and behavioral difficulties; Mediator: Parental self-efficacy					
Toddlers ^1^	−0.15	0.33	−0.98	0.30	
Preschool-aged children ^2^	0.08	0.13	−0.15	0.37	
	--	0.35	−0.30	1.10	0.22

Note. ^1^ Children aged between 18 months and 36 months; ^2^ Children aged between 37 months and 72 months; SE—Standard Error; LLCI—Lowest Limit; ULCI—Maximum Limit.

## Data Availability

Data are available upon reasonable request from the corresponding author.

## Abbreviations

The following abbreviations are used in this manuscript:

WFC	Work–family conflict
PV	Portuguese version
PPSC	Preschool Pediatric Symptom Checklist
PSC-17	Pediatric Symptom Checklist-17
PSS	Parental Stress Scale
HADS	Hospital Anxiety and Depression Scale
MaaP	Me as a Parent (Parental Self-Regulation Scale)

## References

[1] Badawy P., Schieman S. (2020). Control and the health effects of work–family conflict: A longitudinal test of generalized versus specific stress buffering. J. Health Soc. Behav..

[2] Cooklin A.R., Dinh H., Strazdins L., Westrupp E., Leach L.S., Nicholson J.M. (2016). Change and stability in work-family conflict and mothers’ and fathers’ mental health: Longitudinal evidence from an Australian cohort. Soc. Sci. Med..

[3] Cinamon R.G., Weisel A., Tzuk K. (2007). Work-family conflict within the family: Crossover effects, perceived parent-child interaction quality, parental self-efficacy, and life role attributions. J. Career Dev..

[4] Strazdins L., Obrien L.V., Lucas N., Rodgers B. (2013). Combining work and family: Rewards or risks for children’s mental health?. Soc. Sci. Med..

[5] Van den Eynde A., Claessens E., Mortelmans D. (2020). The consequences of work-family conflict in families on the behavior of the child. J. Fam. Res..

[6] Egreja C., Melo S. (2023). Conciliação trabalho-vida pessoal e familiar em profissões sob elevada pressão: O caso dos enfermeiros, polícias e jornalistas. Sociol. Probl. Práticas.

[7] Afonso P., Aleixo O.V., Aleixo R.V., Carvalho D.J., Simões J.A. (2019). Conciliação Trabalho-Família na Profissão Médica: Um Estudo Exploratório. Acta Med. Port..

[8] Borgmann L.S., Kroll L.E., Müters S., Rattay P., Lampert T. (2019). Work-family conflict, self-reported general health and work-family reconciliation policies in Europe: Results from the European Working Conditions Survey 2015. SSM Popul. Health.

[9] Fernandes J.P.L.S. (2023). Desafios na Conciliação Trabalho-Família: Perspetivas de Pais e Mães Trabalhadores/as. Master’s Thesis.

[10] Eurofound (2025). European Working Conditions Survey 2024: First Findings.

[11] Kim Y.M., Cho S.I. (2020). Socioeconomic status, work-life conflict, and mental health. Am. J. Ind. Med..

[12] Lawson K.M., Lee S., Maric D. (2021). Not Just Work-to-Family Conflict, but How You React to It Matters for Physical and Mental Health. Work Stress.

[13] Stansfeld S., Candy B. (2006). Psychosocial work environment and mental health—A meta-analytic review. Scand. J. Work Environ. Health.

[14] Bilodeau J., Mikutra-Cencora M., Quesnel-Vallée A. (2023). Work-family interface and children’s mental health: A systematic review. Child Adolesc. Psychiatry Ment. Health.

[15] Leach L.S., Dinh H., Cooklin A., Nicholson J.M., Strazdins L. (2021). Australian parents’ work-family conflict: Accumulated effects on children’s family environment and mental health. Soc. Psychiatry Psychiatr. Epidemiol..

[16] Achenbach T.M., Rescorla L.A. (2000). Manual for the ASEBA Preschool Forms & Profiles.

[17] Klitzing K., Döhnert M., Kroll M., Grube M. (2015). Mental Disorders in Early Childhood. Dtsch. Arztebl. Int..

[18] Yucel D., Latshaw B.A. (2021). How do mothers’ and fathers’ work–family conflict impact children’s problem behaviors?. J. Fam. Issues.

[19] Dinh H., Cooklin A.R., Leach L.S., Westrupp E.M., Nicholson J.M., Strazdins L. (2017). Parents’ transitions into and out of work-family conflict and children’s mental health: Longitudinal influence via family functioning. Soc. Sci. Med..

[20] Lim M., Pollmann-Schult M., Li J. (2025). Parents’ Work–Family Conflict and Children’s Emotional Well-Being: The Mediating Role of Parenting Behaviors. J. Fam. Issues.

[21] Vahedi A., Krug I., Fuller-Tyszkiewicz M., Westrupp E.M. (2018). Longitudinal associations between work-family conflict and enrichment, inter-parental conflict, and child internalizing and externalizing problems. Soc. Sci. Med..

[22] Trumello C., Ballarotto G., Ricciardi P., Paciello M., Marino V., Morelli M., Tambelli R., Babore A. (2023). Mothers and fathers of pre-school children: A study on parenting stress and child’s emotional-behavioral difficulties. Curr. Psychol..

[23] Bilodeau J., Quesnel-Vallée A., Poder T. (2023). Work stressors, work-family conflict, parents’ depressive symptoms and perceived parental concern for their children’s mental health during COVID-19 in Canada: A cross-sectional analysis. BMC Public Health.

[24] Chen C. (2023). The relationship between parental depression and child internalizing and externalizing problems: The roles of parenting stress and child maltreatment. Front. Public Health.

[25] Huang K.Y., Abura G., Theise R., Nakigudde J. (2017). Parental Depression and Associations with Parenting and Children’s Physical and Mental Health in a Sub-Saharan African Setting. Child Psychiatry Hum. Dev..

[26] Rusu P.P., Candel O.S., Bogdan I., Ilciuc C., Ursu A., Podina I.R. (2025). Parental Stress and Well-Being: A Meta-analysis. Clin. Child Fam. Psychol. Rev..

[27] Santos M.B. (2017). Conflito Trabalho-Família, Stress Parental e Comportamento de Externalização em Crianças em Idade Pré-Escolar. Master’s Thesis.

[28] Vieira J.M., Matias M., Ferreira T., Lopez F.G., Matos P.M. (2016). Parents’ work-family experiences and children’s problem behaviors: The mediating role of the parent–child relationship. J. Fam. Psychol..

[29] Scigala K.D., Fabris M.A.F., Zdankiewicz-Ścigała E., Sikora J., Longobardi C. (2024). Pandemic era maternal alexithymia and burnout as mediated by self-efficacy and resilience. J. Child Fam. Stud..

[30] Albanese A.M., Russo G.R., Geller P.A. (2019). The role of parental self-efficacy in parent and child well-being: A systematic review of associated outcomes. Child Care Health Dev..

[31] Slagt M., Deković M., de Haan A.D., Van Den Akker A.L., Prinzie P. (2012). Longitudinal associations between mothers’ and fathers’ sense of competence and children’s externalizing problems: The mediating role of parenting. Dev. Psychol..

[32] Glatz T., Lippold M., Chung G., Jensen T.M. (2024). A systematic review of parental self-efficacy among parents of school-age children and adolescents. Adolesc. Res. Rev..

[33] Adams A., Golsch K. (2023). Consequences of Work-to-Family Conflicts for Parental Self-Efficacy—The Impact of Gender and Cultural Background in Germany. J. Fam. Issues.

[34] Edvoll M., Kehoe C.E., Trøan A.S., Harlem T.E., Havighurst S.S. (2023). The relations between parent and toddler emotion regulation. Ment. Health Prev..

[35] Lunkenheimer E., Skoranski A.M., Lobo F.M., Wendt K.E. (2021). Parental depressive symptoms, parent-child dyadic behavioral variability, and child dysregulation. J. Fam. Psychol..

[36] Simon P., Nader-Grosbois N. (2023). Empathy in Preschoolers: Exploring Profiles and Age- and Gender-Related Differences. Children.

[37] Netemeyer R.G., Boles J.S., McMurrian R. (1996). Development and validation of work–family conflict and family–work conflict scales. J. Appl. Psychol..

[38] Santos J.V., Gonçalves G. (2014). Contribuição para a adaptação portuguesa das escalas de conflito trabalho-família e conflito família-trabalho. Rev. Electron. Psicol. Educ. Saúde.

[39] Sheldrick R.C., Henson B.S., Merchant S., Neger E.N., Murphy J.M., Perrin E.C. (2012). The Preschool Pediatric Symptom Checklist (PPSC): Development and initial validation of a new social/emotional screening instrument. Acad. Pediatr..

[40] Rocha B., Nunes C. (2021). Psychometric characteristics of the Portuguese version of the Preschool Pediatric Symptom Checklist for children aged 18 to 60 months. Psicologia.

[41] Gardner W., Murphy M., Childs G., Kelleher K., Pagano M., Jellinek M., McInerny T.K., Wasserman R.C., Nutting P., Chiappetta L. (1999). The PSC-17: A brief pediatric symptom checklist with psychosocial problem subscales. A report from PROS and ASPN. Ambul. Child Health.

[42] Pereira A.I., Pires R., Alves S., Barros I. (Faculty of Psychology, University of Lisbon, Lisbon, Portugal) (2020). Checklist de Sintomas Pediátricos [Versão Portuguesa da Pediatric Symptom Checklist].

[43] Berry J.O., Jones W.H. (1995). The Parental Stress Scale: Initial Psychometric Evidence. J. Soc. Pers. Relatsh..

[44] Mixão M., Leal I., Maroco J. (2007). Escala de Stress Parental. Avaliação em Sexualidade e Parentalidade.

[45] Algarvio S., Leal I., Maroco J. (2018). Parental Stress Scale: Validation study with a Portuguese population of parents of children from 3 to 10 years old. J. Child Health Care.

[46] Louie A.D., Cromer L.D., Berry J.O. (2017). Assessing parenting stress: Review of the use and interpretation of the parental stress scale. Fam. J..

[47] Snaith R.P., Zigmond A.P. (1994). The Hospital Anxiety and Depression Scale.

[48] Pais-Ribeiro J., Silva I., Ferreira T., Martins A., Meneses R., Baltar M. (2007). Validation study of a Portuguese version of the Hospital Anxiety and Depression Scale. Psychol. Health Med..

[49] Hamilton V.E., Matthews J.M., Crawford S.B. (2015). Development and preliminary validation of a parenting self-regulation scale: “Me as a Parent”. J. Child Fam. Stud..

[50] Marques T., Cadete, Barros L., Pereira A.I. (Faculty of Psychology, University of Lisbon, Lisbon, Portugal) (2015). Escala de Autorregulação Parental.

[51] Marques T., Pereira A.I., Barros L., Roberto M.S. (2020). Estrutura fatorial da escala “Me as A Parent” numa amostra comunitária de mães portuguesas. Psicologia.

[52] Cohen J. (1988). Statistical Power Analysis for the Behavioral Sciences.

[53] Hayes A.F. (2013). Introduction to Mediation, Moderation, and Conditional Process Analysis: A Regression-Based Approach.

[54] Hayes A.F. (2022). Introduction to Mediation, Moderation, and Conditional Process Analysis: A Regression-Based Approach.

[55] Bronfenbrenner U. (1979). The Ecology of Human Development: Experiments by Nature and Design.

[56] Moreira H., Fonseca A., Caiado B., Canavarro M.C. (2019). Work-family conflict and mindful parenting: The mediating role of parental psychopathology symptoms and parenting stress in a sample of Portuguese employed parents. Front. Psychol..

[57] Simon P., Nader-Grosbois N. (2021). Preschoolers’ empathy profiles and their social adjustment. Front. Psychol..

